# Attention 3D U-Net with Multiple Skip Connections for Segmentation of Brain Tumor Images

**DOI:** 10.3390/s22176501

**Published:** 2022-08-29

**Authors:** Jakhongir Nodirov, Akmalbek Bobomirzaevich Abdusalomov, Taeg Keun Whangbo

**Affiliations:** 1 Department of IT Convergence Engineering, Gachon University, Sujeong-Gu, Seongnam-Si 461-701, Gyeonggi-Do, Korea; 2 Department of Computer Engineering, Gachon University, Sujeong-Gu, Seongnam-Si 461-701, Gyeonggi-Do, Korea

**Keywords:** brain image, deep learning, CNN, U-Net, pretrained MobileNetV2, attention modules, skip connections

## Abstract

Among researchers using traditional and new machine learning and deep learning techniques, 2D medical image segmentation models are popular. Additionally, 3D volumetric data recently became more accessible, as a result of the high number of studies conducted in recent years regarding the creation of 3D volumes. Using these 3D data, researchers have begun conducting research on creating 3D segmentation models, such as brain tumor segmentation and classification. Since a higher number of crucial features can be extracted using 3D data than 2D data, 3D brain tumor detection models have increased in popularity among researchers. Until now, various significant research works have focused on the 3D version of the U-Net and other popular models, such as 3D U-Net and V-Net, while doing superior research works. In this study, we used 3D brain image data and created a new architecture based on a 3D U-Net model that uses multiple skip connections with cost-efficient pretrained 3D MobileNetV2 blocks and attention modules. These pretrained MobileNetV2 blocks assist our architecture by providing smaller parameters to maintain operable model size in terms of our computational capability and help the model to converge faster. We added additional skip connections between the encoder and decoder blocks to ease the exchange of extracted features between the two blocks, which resulted in the maximum use of the features. We also used attention modules to filter out irrelevant features coming through the skip connections and, thus, preserved more computational power while achieving improved accuracy.

## 1. Introduction

Brain tumors are masses of abnormally growing cells in the brain. They can originate in the brain, which would be primary brain tumors, or migrate from other parts of the body, which would be secondary (metastatic) brain tumors. There are different types of brain tumors such as noncancerous (benign) and cancerous (metastatic) ones. Currently, in the United States alone, 700,000 people live with primary brain tumors, and more than 85,000 were diagnosed in 2021. The survival rate of patients with brain tumors depends on various factors, such as the age of the patient, as indicated in [1], which shows that survival rate of a year among patients ages 55–64 is 46.1%, while for patients ages 65–74, it is only 29.3%. In addition, an early diagnosis of tumors also plays vital role on survival rate, as shown in [2]. Recently, automated brain tumor detection models have become more accurate and helpful with the continued development of machine learning and deep learning techniques. This makes it possible to reduce the cost of brain tumor detection technology and make it available to more people. As a result, detection of tumors in more people on time has become possible, improving their survival rate. Therefore, many studies have been conducted to develop more accurate brain image segmentation models, and these models dramatically outperform traditional tumor detecting methods. U-Net, Res-Net [3], and DenseNets [4] were previously developed, but they segmented only 2D input images, causing them to miss additional contextual information between slices. The rapid development of machine learning and deep learning methods has led to the development of fast, accurate, and inexpensive methods for creating 3D volumetric data from 2D brain image slices. This enables us to work in the 3D domain. As a result, 3D U-Net, VNet, and other 3D models have emerged. They can extract a higher number of features than the 2D models such as U-Net. U-Net-based models are the most popular models in the medical field because of the high accuracy and efficiency of their deep network architecture with encoder and decoder blocks, which effectively extract features in global and local fields. Skip connections play an important role as they are responsible for passing extracted features to the decoder block from the encoder block to detect the tumor. In recent years, different studies using U-Net++ [5] and U-Net3+ [6] have used combinations of skip connections. Moreover, attention models [7] have also shown huge potential and have been actively used in vision tasks. In the medical field, especially with U-Net, multiple studies have been conducted to increase the accuracy of the model with attention models filtering out irrelevant features from the input images to save computational power and accelerate the model. For example, in [8], the authors proposed a novel attention gate method based on U-Net [9] architecture. In their research work, the authors stated that the attention module suppresses the irrelevant regions of the input image, while highlighting the salient features. They also stated that a model integrated with attention modules can perform with minimal computational overhead, while increasing the sensitivity and prediction accuracy of the model.

In this study, we aim to increase the accuracy of the model, while tracking its use of computational power. For this purpose, we used the following components: MobileNetV2 blocks to help maintain the desired architecture size given that 3D segmentation models usually require more power than their 2D alternatives; additional skip connections to improve the flow of features from the encoder to the decoder block, resulting in the creation of a higher number of unique features for tumor detection; and an attention model to filter and pass relevant features coming through the skip connections, saving computational power and time for computation.

In summary, the main contributions of the research work are as follows:

- A fully automated brain tumor segmentation model has been developed for classifying brain tumors in human brain images that was trained using the BRATS 3D Brain Image dataset. Our model achieved the best inference time and was easy to deploy compared to other methods.
- We have created a power-efficient computing model using the MobileNetV2 backbone. Due to the complexity of preserving the computational power of 3D deep learning architectures, the training process requires high computational power. In our case, we were able to train it by decreasing the number of parameters, after applying the MobileNetV2 backbone in the encoder of the architecture.
- Our model is also the most time efficient during the training process. The pretrained MobileNetV2 backbone that we used in our architecture, for the encoder blocks, assists the model to converge faster than any other model we compared it to.
- Our architecture had the best output out of the other state-of-the-art architectures we compared it to in the same technical environment. This result was attained by applying revised skip connections, which create the free flow of previously created features between the encoder and decoder blocks. In addition, the attention modules filter the irrelevant features to prevent diminishing accuracy confidence.

The remainder of this paper is organized as follows. Section 2 briefly discusses the recent studies related to our research. In Section 3, we present the proposed method. We then present the results and provide a comparison of the model with other models as well as the relevant statistics in Section 4. Section 5 highlights certain limitations of the proposed method. In Section 6, we discuss the implications of our work and conclude the paper.

## 2. Related Work

Research studies that use computer vision technology for brain image segmentation started a long time ago, and, thus, a large number of studies have been conducted so far. Different types of tumor segmentation applications exist today.

### 2.1. Traditional Image Processing Methods

In this method, certain connected image regions are extracted from an input image. These regions are connected based on the pixel or voxel intensity of the image region. This is accomplished by selecting certain initial points, locating them in the region of the image, and then extracting the regions with points that are connected through similar intensity values. For example, in [10], a localized active contour model was employed, which is a region-based segmentation method, and it was successfully applied to segment brain tumors. Moreover, the thresholding method categorizes a certain region based on the pixel or voxel information of an image with similar intensity values and converts the input image to a binary image. The output is then compared to other thresholds. Then, the authors used a selective level set formulation, which allows the proposed method to capture the region of interest inside an image. This method greatly increased the model efficiency in terms of accuracy and computing cost. In [11], a thresholding method was used to segment brain image tumors. First, the image was enhanced; subsequently, morphological operations were applied to identify boundaries of the object in the image, and then pixel subtraction was performed to segment a tumor. The main advantage of the method is its easy implementation for practical problems, with more efficiency. However, the accuracy is not compatible with newer methods.

### 2.2. Traditional Machine Learning Methods

Different machine learning techniques such as classification and clustering methods have been used to detect brain tumors. Typically, classification techniques classify the given input data. Regarding brain images, the input images can be classified as tumor-related or non-tumor-related, depending on the multidimensional feature spaces, which can be obtained by processing several brain image modalities. This model should be trained with labeled images first. For example, in [12], a k-nearest neighbor classifier was used to classify tumors as either benign or malignant. In the clustering method, unlabeled input images are clustered into classes based on the similarity of pixel features, without training, as is usual in unsupervised machine learning. Despite the lower accuracy compared to the state-of-the-art deep learning models, this method is very efficient when there are not enough data available, which is very common in the medical field. In [13], a k-means clustering algorithm was used to cluster brain tumors as the base model, which is more reliable if there are few data available, though there is slightly reduced accuracy.

### 2.3. Deep-Learning-Based Methods Using 2D Images

Recently, deep learning has started to outperform traditional state-of-the-art machine learning techniques. Deep networks, which create high semantic features, function more effectively compared to manually created features in traditional machine learning algorithms. As a result, deep learning algorithms are becoming popular, making traditional machine learning algorithms obsolete for some practical problems. Recently, in the medical field, deep-learning-based vision models have especially become hugely popular because of their accuracy. An early study [14] on brain image segmentation using deep learning, used a convolutional neural network (CNN) model to segment brain tumors, gliomas in particular. This study used small 3 × 3 kernels to create a deeper learning model and help overcome overfitting problems by using intensity normalization techniques. The deep learning CNN algorithm used was more efficient than traditional machine learning algorithms, in terms of accuracy when there are more data available. In [15], a CNN model was also used that was different from traditional machine learning algorithms. Using fully connected layers, a speed increase of 40 times as compared to traditional methods was achieved, which was a breakthrough in the segmentation field. The model required more computational power for a dataset with bigger numbers compared to the traditional machine learning algorithms. Subsequently, competitive studies on CNN models have been conducted and published.

In [16], they have used CNN-based deep learning architecture to classify fetal ultrasound images. In particular, certain types of models with encoder and decoder blocks, such as U-Net, have become widespread in the medical research community because of their high efficiency in extracting semantic features from medical images. Originally, U-Net was constructed in the encoder and decoder bases, and skip connections were used to pass features generated from the decoder to the encoder. These features help the model to detect tumors and also solve the gradient vanishing problem, which is a common problem among deep networks owing to its extreme network depth, and it is highly unlikely to backpropagate the output of the network to calculate loss. Skip connections directly exchange features in upper networks; therefore, the features flow easily through the blocks, and the training output can be effectively backpropagated. Decoder blocks usually reconstruct labeled images using extracted features. Since U-Net was originally proposed, many studies have been conducted on different fields using the architecture as a base model. For example, in [17], they have created a U-Net-based model for land field segmentation, which shows the use case of U-Net in different fields. In addition, in [18], the U-Net model was used as a base, and a new architecture called Dense-Res-Inception Net was created. This architecture contains convolution blocks with dense connections and deconvolution blocks with residual inception models that outperform U-Net in the multiclass segmentation of different brain images taken under three different conditions.

In [19], additional up-skip connections were proposed to enhance the flow of information between the encoder and decoder blocks containing inception modules in each block. This novel architecture was also based on the U-Net model. The main advantage of the model is the use of inception structure to address segmentation problems, which achieves high performance while maintaining computational efficiency. However, due to the increasing number of parameters, the model was trained with more difficulty and slower testing. In [20], an attention gate module was used in a U-Net-based architecture with residual blocks, and a novel architecture, called the AGResU-Net, was created to highlight low-level features and filter noisy ones. On the one hand, residual modules enhance the ability of feature extraction and expression and contribute to the classification in the process of down sampling. On the other hand, attention gates pay more attention to small-scale tumors and obtain more information about the location of small-scale tumors, so that the up-sampling process is helpful to restore the location information of small-scale tumors. However, the authors used 2D convolution rather than 3D convolution, which is more efficient with a 3D MRI dataset. In [21], the authors created a new architecture called A-DenseU-Net, which is based on U-Net architecture, with attention modules used along the dense blocks and atrous convolution applied instead of regular convolutions. The method also uses residual blocks, and it improves the outcome of the colorectal polyp segmentation compared to other state-of-the-art methods. The model obtained good results with small datasets.

### 2.4. Deep-Learning-Based Methods Using 3D Images

The rise of computer vision technology has facilitated the creation of 3D MRI data, because more applications exist that create the available 3D data. In [22], computer vision techniques such as the marching cubes algorithm, bilinear interpolation, bicubic interpolation, and VTK tools were used to create 3D volume reconstruction applications from MRI slices. In [23], a deep learning model, specifically, a generative adversarial network with 3D registration methods, was used to reconstruct 3D volumes. These methods ease the creation of big data for 3D CNN segmentation models. As a result, 3D brain image segmentation models have become popular, and a higher number of them have been proposed based on previous 3D architectures, such as 3D U-Net [24] and V-Net [25]. For example, in [26], a 3D architecture with residual learning was proposed, and it was applied with dilated convolution as a successful brain image segmentation model. The architecture was designed to segment the brain into various regions including cerebrospinal fluid, white matter, and gray matter. Unlike traditional segmentation methods in medical imaging that are graph-theory-based or atlas-based techniques, this work was directed towards the use of a 3D deep learning segmentation algorithm. In [27], they have proposed new 3D architecture called VoxRes-Net for brain image segmentation. The architecture was built on top of residual blocks. They converted a 2D version into a 3D version with some revised modules and algorithms such as the auto-context algorithm [28], to integrate low-level and context details by fusing low-level features with implicit-shape details. The model borrows the spirit of deep residual learning to tackle the task of object segmentation from volumetric data. In [29], they developed a 3D version of Fully Convolution Neural Network to effectively segment brain tissues, while integrating coarse and dense feature maps to classify tint tissue regions more clearly. In addition, they also proposed an extra fusion model to provide better fusion of the feature maps, to connect the aggregating layers in a better condition. They used batch normalization to speed up the convergence of the networks, especially when hierarchical feature aggregations occur. By integrating multimodal information, the authors boosted the segmentation performance. In [30], they proposed a new transformer-based method for 3D medical image segmentation. The method is efficient for extracting local and global characteristics compared to other state-of-the-art works. In this method, the authors designed a combination of transformer structure and CNN to achieve better performance, and they also designed a module, ETrans, to enhance detail feature extraction. This module was used to extract local detailed features, so that the model also has strong segmentation capabilities for categories that occupy a small proportion of the image. However, due to extensive use of the transformer structure, which caused the insufficient performance when segmenting the edges. In [31], the authors proposed a transformer-based U-Net shape architecture that is computationally efficient to handle large-sized 3D volumes and learns representations that are robust against artifacts. The model achieved comparable good results, while the accuracy is still insufficient to deploy for real-world use. In [32], the authors mentioned the high computational-power resources required for 3D image segmentation architectures and analyzed data parallelism techniques to accelerate training time and computing power consumption, showing potential techniques for scaling parallel computing on GPUs and nodes with the implantation of a 3D U-Net segmentation model on a brain image dataset. However, further research still needs to be conducted in this field.

## 3. Proposed Architecture

In our proposed architecture, we used 3D U-Net as a base, the novel skip connection architecture in U-Net 3+ as a low-level feature extractor, an attention gates module as a noise filter, and pretrained MobileNetV2 as a computational power preserver. In general, these were used as the core of our model, for creating a novel attention 3D U-Net with multiple skip connections for the segmentation of brain tumor images.

### 3.1. Overview

**Figure 1:**
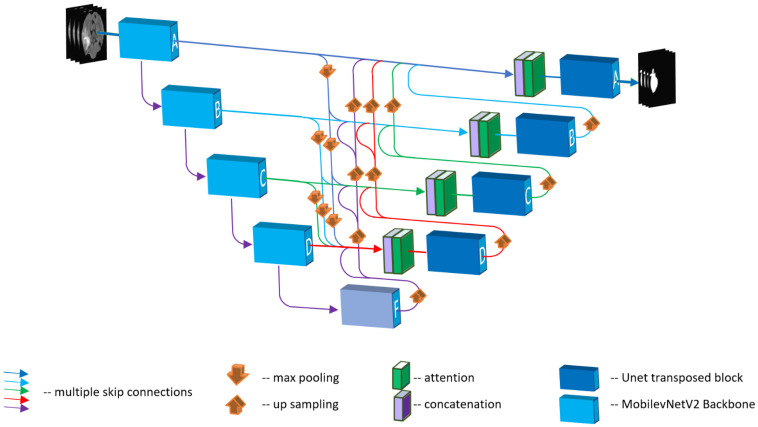
Block diagram of the architecture of our proposed model; MobileNetV2 backbone is used as an encoder. Transposed convolution is performed for up-sampling between levels.

Our architecture was constructed at the base of a 3D version of U-Net. This contains the main decoder and encoder blocks. The main differences of our proposed model from the original 3D U-Net are the use of the MobileNetV2 [33] backbone in the encoder as well as the multiple skip connections and attention modules. Figure 1 shows the entire structure. Our model accepts 3D MRI images with a size of 240 × 240 × 155 voxels. Then, on the left, the encoder block performs convolution operations, ReLU activations, and batch normalizations. In a total of five steps, the size of the input image gradually decreases, while the number of channels increases until the last encoder block F. In this last encoder stage, the convolution block acquires the input and processes it as previous blocks, passing it to the decoder blocks with up-sampling by using transposed convolution. Low-level sematic features are extracted from the input image in the upper blocks of the encoder, and the high-level features are extracted in the lower blocks. The decoder then performs the opposite operation, i.e., using the up-sampling technique, the original size of the image is reconstructed. In this process, skip connections provide the network with high-level sematic image information and help backpropagate the result to calculate the loss. In our model, we added skip connections to optimize the flow of features between the encoder and decoder blocks. Our attention gate modules filter out the noisy features and only pass relevant features, to save significant computational power and improve accuracy.

### 3.2. MobileNetV2

**Figure 2:**
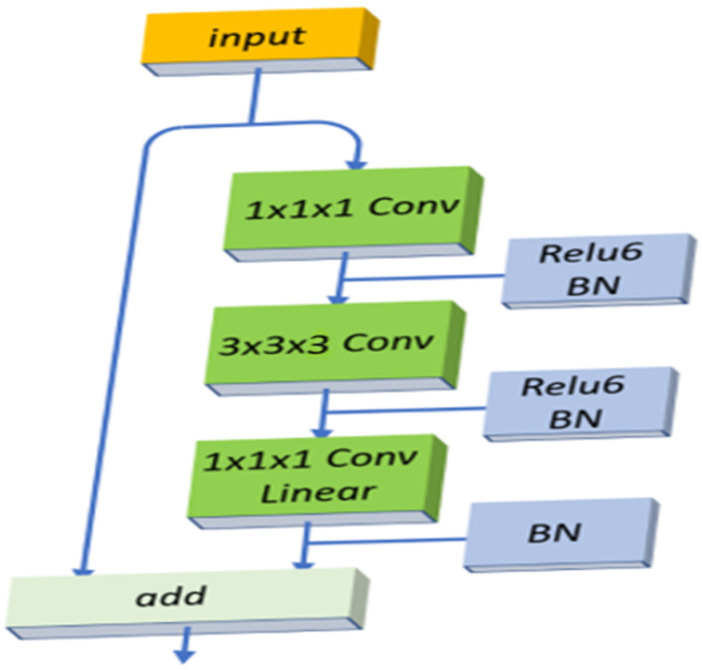
The 3D version of modified MobileNetV2 backbone is used as an encoder block.

We used a 3D version of the MobileNetV2 backbone as the encoder blocks in our model. This architecture is an improved version of MobileNetv1 [34], with linear bottlenecks between layers and shortcut connections between bottlenecks, as shown in Figure 2.

First, the block takes a set of features with depth, width, and height to do a 1 × 1 × 1 convolution with ReLU6 and batch normalization. In the second layer, the input is processed with a 3 × 3 × 3 depth-wise convolution to convolve three RGB channels using ReLU6 and batch normalization, again in the original architecture. However, in our case, we use volumetric data. Therefore, we use 3D depthwise voxels of our 3D input image, since our data do not contain three RGB channels. In the last layer of the block, another 1 × 1 × 1 convolution is applied without an activation function. The output is then added to the previous input. This block helps our model maintain a low size, as 3D convolutions require significant computational power. This enables the extraction of as many features as possible from the input image in each step of the low-cost, depth-wise convolution. Moreover, we applied the transfer learning technique and used MobileNetV2 with 3D pretrained weights. This method helped our model to converge faster and smoother than other models, while slightly improving the accuracy.

### 3.3. Multiple Skip Connections

We used a 3D version deep model that allows modules to extract more high-level features with a high accuracy, but calculating the loss depends on the backpropagation algorithm used by the network, which is less efficiently used by deeper networks. To solve this problem, skip connections have been successfully proposed for the Res-Net and U-Net architectures. In Res-Net, the skip connections skip over one or more layers with their related operations to solve the vanishing problem and diminishing accuracy. With the previous layer’s output x_0,_ traditional neural network architectures first perform summing the input and weights and then activate the input with the activation function. Usually, the process repeats two times, as shown in Equation (1).

(1) $$\begin{array}{c} z_{1}=w_{1}x_{0}+b_{1}\to \;x_{1}=\mathrm{ReLU}\left(z_{1}\right)\\ z_{2}=w_{2}x_{1}+b_{2}\to \;x_{2}=\mathrm{ReLU}\left(z_{2}\right) \end{array}$$

With the skip connections, we do the same process but with one more operation. We pass the x_0_ and add it along with $z_{2}$ in the second activation function, as shown in Equation (2).

(2) $$x_{2}=\mathrm{ReLU}\left(z_{2}\right)$$

The activation function outputs previously received two inputs directly, if all the values are positive. If the value of $z_{2}$ is negative, then it only outputs $x_{0}$, and the output will be as follows, in Equation (3).

(3) $$x_{2}=\mathrm{ReLU}\left(0\right)$$

In contrast, without skip connections, we have only $z_{2}$ with a 0 value, which will be deactivated. Further, this can lead to the problems we mentioned above. This skip connection operation allows the network to bypass some outputs with a 0 value and perform convolution operations in deeper networks without loss. In U-Net architecture, almost the same operations are required. The output of previous encoder layers is passed to the decoder layers rather than the next encoder layers. Moreover, instead of an addition operation in U-Net they used a concatenation operation. Subsequently, redesigned versions of skip connections were proposed as U-Net++ and U-Net3+. In our study, we used the skip connection architecture of U-Net3+ to improve the feature flow and extraction of low-level features. At the first block, we take the output as a skip connection and share it with the bottom three decoder blocks. From the second encoder block, we share the output to the bottom two decoder blocks, and the process continues until the fourth encoder block is reached. The same process is applied to the decoder blocks. This time, we go from the bottom decoder block until the last decoder block. The outputs are shared with the upper blocks.

**Figure 3:**
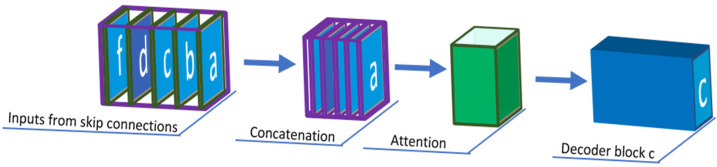
Output from encoder blocks A, B, C, and F and decoder block D are passed through skip connections to decoder block C. In between, the past features are concatenated and then filtered through attention module.

In Figure 3, we took a single decoder block, C, as an example and explain how the block is receiving multiple inputs from the other encoder and decoder blocks. Those multiple skip connections prevent losing the fine features extracted in each decoder and encoder block, passing the original features to every convolution block to extract more unique features. Each decoder block in our network incorporates both smaller and same-scale feature maps from encoder blocks and larger-scale feature maps from decoder blocks, which capture fine-grained details and coarse-grained semantics in full scales.

### 3.4. Attention Models

In our study, the attention module was one of the main modules. Recently, the attention module [35] was proposed and has become popular, especially in sequence-related tasks such as language learning. This fundamental attention mechanism consists of a query, keys, values, and a score function. The query indicates which feature vectors are to be paid attention to. Keys are also feature vectors, but they are the features that later help to identify elements with their query. Values are given to each input element; therefore, the average is calculated if the element is the one to be paid attention to. The score function is the primary function of the attention gate. It takes the query and key as inputs and outputs the score of each input. The last output is then processed with a value to determine how much attention is to be paid to each element. Overall, the attention mechanism is the weighted average of the elements, with weights that are calculated based on the key and query. After successfully purposing the attention module in language-learning sequential tasks, the attention gate, called Attention Gates, was successfully proposed to the CNN domain.

**Figure 4:**
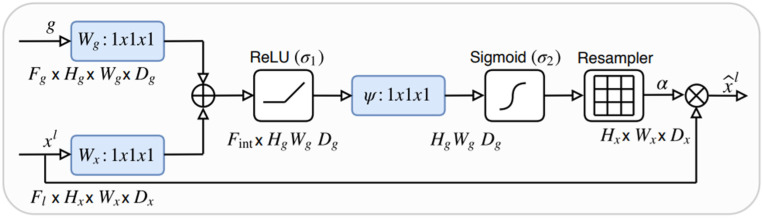
Architecture of attention module.

Figure 4 presents the main structure of the module. First, two input feature maps are obtained: gating vectors g and x, which are obtained from output layer l by sequentially applying a linear transformation. Thereafter, a 1 × 1 × 1 convolution is performed for both the vectors, and they are concatenated with the applied ReLU activation function. Then, the same convolution is performed a second time with a sigmoid activation. Afterwards, the output is processed with trilinear interpolation to restore the image size. Finally, the final output and previous input x are concatenated. This attention gate module is applied between the decoder and encoder blocks in our proposed architecture. They receive features from the multiple skip connections, and the received features are processed to filter local features and emphasize low-level features. This enables the model to automatically choose the relevant regions of the input image, extract only the relevant features, and pass them along for further processing.

## 4. Experimental Results

In this section, we present some experimental results obtained by implementing our model and using other tools to analyze them.

### 4.1. Dataset

**Figure 5:**
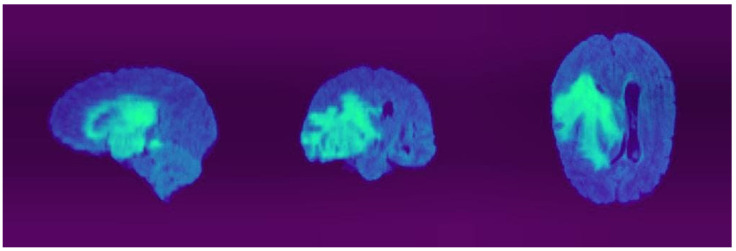
The 3D MRI from BRATS-2020 brain image dataset.

We used the BRATS-2020 brain image dataset [36,37,38] to train our model. This dataset consists of clinically acquired multimodal magnetic resonance images (MRIs) of glioblastomas (GBM, HGG) and low-grade gliomas (LGG). The dataset was provided for the training, validation, and testing of the 3D MRIs. All images were in the Nifty format. There are native (T1), post-contrast T1-weighted (T1Gd), T2-weighted (T2), and T2 fluid-attenuated inversion recovery (T2-FLAIR) volumes acquired by different institutions within the dataset. All images were manually segmented by experienced neuroradiologists. In Figure 5, one of the 3D image examples can be seen. The dataset contains 367 MRI images for the training set and 123 MRI images for the validation set. Each image has 5 modalities and a 3D volume size of 240 × 240 × 155 pixels.

### 4.2. Evaluation Metrics

When evaluating typical deep learning models, we normally categorize predictions into four categories: true positives, false positives, true negatives, and false negatives. Nevertheless, for dense prediction tasks in image segmentation, it is not directly clear what is assigned for “true positives” or, more generally, how to evaluate predictions. Additionally, medical image segmentation is a critical image processing stage in medical field. Comparing images to assess the quality of segmentation is an essential part of measure progress in this area of study. In this section, we discuss and explain several evaluation metrics we used in our work. 

We used the following evaluation metrics: precision, recall, Dice score [39], and Hausdorff distance [40]. Precision and recall measurements were calculated using the following formulas, as in our earlier studies [41,42,43,44], with true positive (TP), false negative (FN), and false positive (FP) values.

(4) $$\mathrm{Recall}=\mathrm{TP}/\left(\mathrm{TP}+\mathrm{FN}\right)$$

(5) $$\mathrm{Precision}=\mathrm{TP}/\left(\mathrm{TP}+\mathrm{FP}\right)$$

Precision refers to the relevant cases among the retrieved cases, whereas recall refers to the fraction of the retrieved cases among the relevant cases. The Dice score is the Dice similarity coefficient, which is an overlap measure that calculates the differences between the ground truth and segmentation result, as shown by the following formula:

(6) $$\mathrm{Dice}\;\mathrm{score}=2*\frac{\left|Y^{\prime }\cap \;Y\right|}{\left(\left|Y^{\prime }\right|+\left|Y\right|\right)}=\frac{\mathrm{TP}}{\left(\mathrm{TP}+0.5*\mathrm{FP}+0.5*\mathrm{FN}\right)}$$

**Figure 6:**
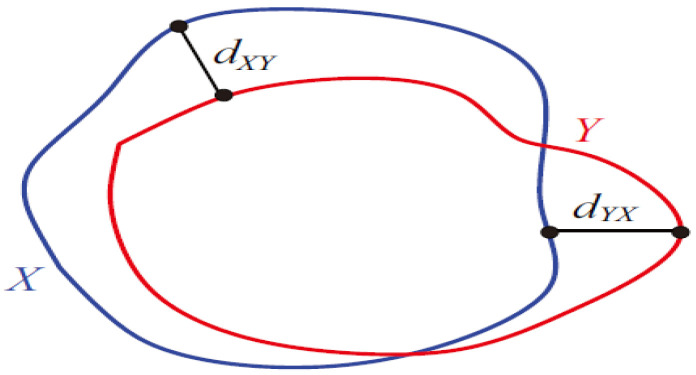
Hausdorff distance.

Here, Y′ is the prediction, and Y is the actual labeled image. The Hausdorff distance is the surface distance that measures the maximum distance from the point sets to the nearest point in other sets. Figure 6 shows the clear picture of Hausdorff distance. The formula for calculating the Hausdorff distance is as follows:

(7) $$D_{H}\left(X,Y\right)=max\left\{sup_{x\in X}\;inf_{y\in Y}\;d\left(x,y\right),\;sup_{y\in Y}inf_{x\in X}d\left(x,y\right)\right\}$$

Here, sup is the least upper bound, inf is the greatest lower bound, and d is the Euclidean matrix.

### 4.3. Implementation Details

To implement the model, we used the PyTorch framework. The model used 3D brain MRIs with a size of 240 × 240 × 155 voxels. During the training process, we tried different hyperparameter values to learn the optimal ones that work the best for our architecture. We created a table and recorded the values with their effect on performance output. After several attempts, we discovered the best values. We trained our model for 200 epochs with a batch size of 2. We tried with bigger batch sizes, but our computational power could not handle them. We also tried to decrease parameter size by decreasing future map, but it affected model’s accuracy negatively. For the optimizer, we tried Adam, Adamax, NAdam, SGD, and Adagrad optimizers and found Adam had the best performance. We set the learning rate to 0.01 and the learning rate scheduler at a factor of 5, which decreased if the metric stopped improving for 5 consecutive epochs. We also used different non-linear activation functions such as ELU, ReLU, Hardtanh, and LogSigmoid. At the end, we found out the ReLU activation function is most ideal nonlinear activation functions for our model. We used a binary cross-entropy loss function to calculate the loss.

### 4.4. Quantitative and Qualitative Results

**Figure 7:**
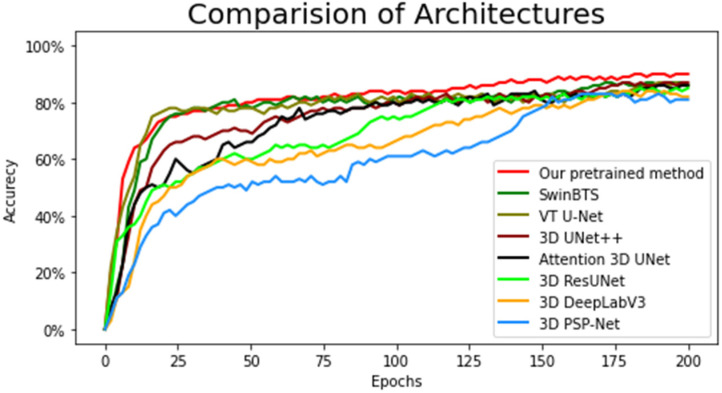
Comparison of selected architectures.

To compare our results, we picked seven state-of-the-art architectures, SwinBTS, VT U-Net, U-Net++, Attention U-Net, 3D U-Net Res-U-Net, DeepLabV3 [45], and PSP-Net [46] and compared their results with ours in the same environment to obtain fair evaluation results. For all models, we used the same input image of the same size. Table 1 shows the evaluation results for all models. Our proposed architecture achieved the best performance among the chosen architectures with attention modules, multiple skip connections, and pretrained MobileNet blocks in the encoder. The results of our proposed architecture recorded a Dice score of 0.8974, Hausdorff distance of 5.76, recall of 0.8863, and precision of 0.9087. Basic 3D U-Net performed poorly among the chosen architectures, with a Dice score of 0.8316. Compared with other architectures, basic 3D U-Net has a simple architecture because it is the oldest architecture among the four models. One of the selected architectures, 3D U-Net++, showed better results than other architectures. Moreover, we have tested new transformer-based architectures and compared them. They had better results with fast convergence rate. However, they still lack accuracy compared to our proposed method. For first steps, the SwinBTS architecture converged much better, and, for later steps, our method showed better accuracy. The VT U-Net architecture also had good convergence rate, but, overall, it achieved slightly lower result. In Figure 7, we visualized the Dice score outputs. From the given graph, it is clear that our proposed method outperforms the other selected architectures, while converging faster and smoother than any other architectures.

**Figure 8:**
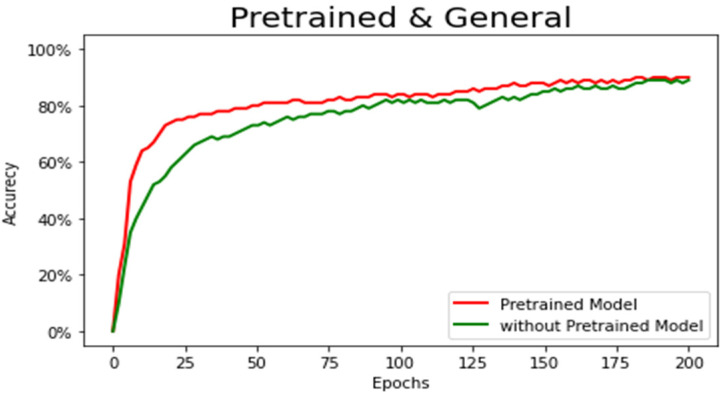
Comparison of our model with and without pretrained weights.

Figure 8 compares the dice score of our model with and without pretrained weights. The visualized output shows that the model with pretrained weights had dominance. It converged faster and smoother compared to the model without weights. Moreover, there were slight improvement in accuracy.

**Figure 9:**
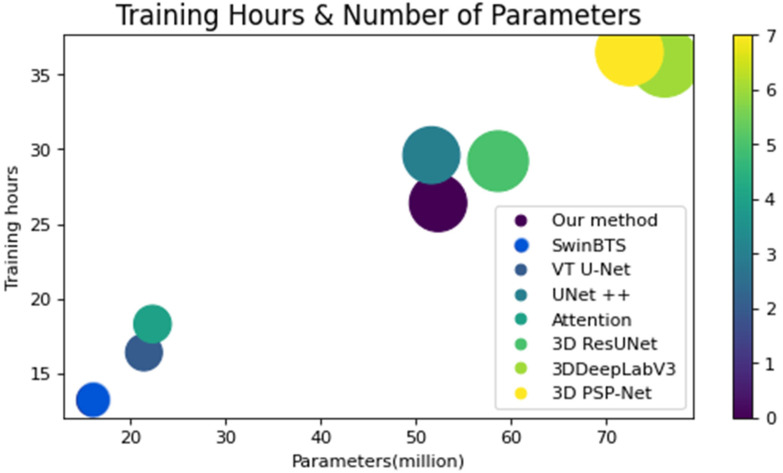
Comparison of Dice score and the number of parameters.

**Figure 10:**
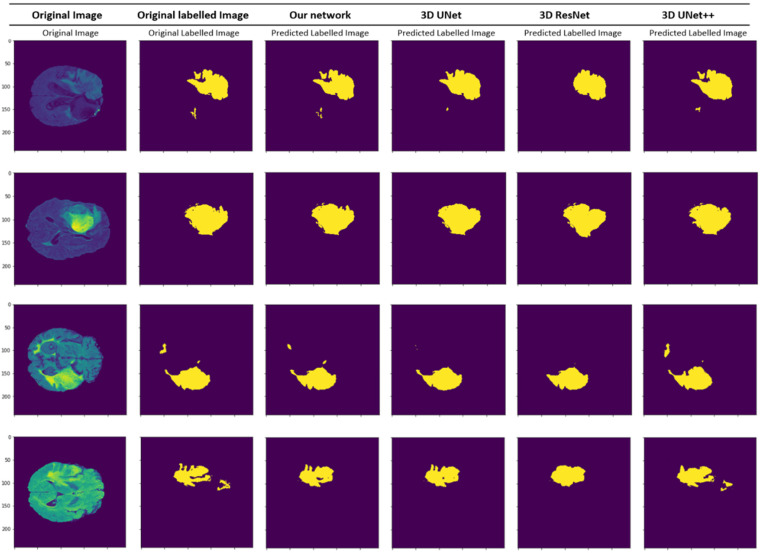
Qualitative segmentation results of various models on the BRATS-2020 dataset. Experimental results show that our method produces better segmentation masks than other state-of-the-art networks.

In Table 2, the training time, prediction time, and number of parameters are shown. Our model spends around 26 h for training and 55.7 s for prediction time, with 52.3 million parameters. New transformer-based architectures achieved better inference time compared to other architectures, due to their computational cost-efficient architectures, while they still perform below our proposed model. Figure 9 shows a clear picture of the relationship between Dice score and the number of parameters of selected architectures. It shows our model lies in the middle of the parameter range, while doing the best in the accuracy range. Figure 10 proves, with qualitative results, the visual results of segmented brain tumor per model. In this figure you can see the difference of models and the clear dominance of our model. 

Additionally, we experimented by removing each block in our architecture, with implementation. The results are recorded in Table 3. Those quantitative output results show that each block plays vital role in our model. The pretrained weights affected our model positively and showed better outputs in terms of convergence speed and accuracy. Without the MobileNetV2 block, we were not able to run the model, as it requires large amount of computational power (which we do not have), while decreasing the number of channels leads to poor quality.

## 5. Limitations

**Figure 11:**
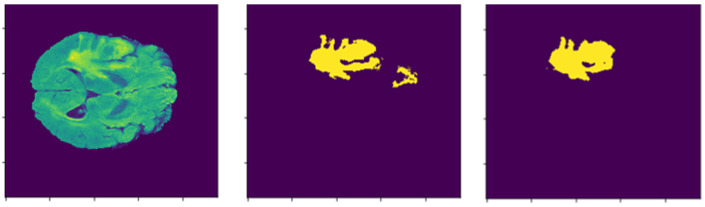
Lack of accurate segmentation.

After analyzing the experimental results of our model, we found that there are still the qualitive limitations that should be improved, by applying a more complex and deeper network structure. Figure 11 shows that our method missed some parts of the label, while segmenting the tumor. Using the MobileNetV2 backbone affects the future extraction process and decreases the quality, as the architecture is lighter than the original U-Net encoder blocks. To solve this problem, we need to alter the MobileNetV2 backbone; however, our 3D network structure requires a substantial amount of computational power, which forced us to use the MobileNetV2 backbone to decrease the number of parameters. We were able to run our network architecture, only after applying the lighter backbone. In order to solve the problem, we are planning some other computational power-efficient architectures, such as zero-shot [47], active appearance model [48] and transformers with convolution [49], for the further development of our model.

## 6. Discussion and Conclusions

In this paper, we describe our proposed model. The experimental results demonstrate that our model has the best performance compared to the other models, for every evaluation method. The multiple skip connections with attention modules assist our model in extracting more low-level features, the MobileNetV2 backbone maintains the architecture at the desired size, and the pretrained weights assist the model to converge faster and smoother with slightly better accuracy. Among the chosen architectures, transformer-based architectures showed the advantage in converging, due to their state of architecture. However, extensive use of transformer architecture led to lower output accuracy, due to a lack of edge feature extraction. Moreover, 3D U-Net++ performed competitively, which is closer to our proposed architecture, because of its more sophisticated architecture compared to the basic 3D U-Net and 3D Res-Net. During the training process we used different approaches to design the best architecture. We found that our model performed best with the ReLU activation function, whereas with the MobileNetV2 backbone, we used ReLU6. Among the loss functions, the binary cross-entropy loss function best suited our proposed model. We also attempted to increase the batch size; however, owing to insufficient computational power, this could not be achieved. We attempted to crop images with a smaller voxel size, which resulted in a worse output. We applied different data augmentation techniques, such as random image filtering and random histogram matching; however, these attempts did not improve the performance of our model.

For future work, we will use our model to conduct research on uncertainty estimations, which are crucial in the segmentation of medical images for the further processing of tumors. Additionally, we are considering modifying our model with transformers, because in recent computer vision research the state-of-the-art segmentation performances have been greatly improved using this architecture. Moreover, the attention to data augmentation is insufficient. In the medical image analysis field, a data shortage is always influencing model performance, due to the difficulty of obtaining and labeling data [50,51,52,53]. The data augmentation method is an important research field to solve this problem, though many works have not mentioned this much. Only some traditional data augmentations and few newly proposed data augmentations are observed, for instance, the GAN-based method. More sophisticated and advanced data augmentation methods should be taken into consideration [54,55].

## Figures and Tables

**Table 1:** Comparison results with other existing methods.

*Methods*	*Dice*	*Hausdorff*	*Recall*	*Precision*
*Our method*	0.8854	5.90	0.8784	0.8925
*Pretrained*	0.8974	5.76	0.8863	0.9087
*3D U-Net*	0.8215	6.97	0.8132	0.8299
*SwinBTS*	0.8764	6.12	0.8733	0.8795
*VT U-Net*	0.8745	6.97	0.8699	0.8790
*3D Res-Net*	0.8567	6.81	0.8495	0.8641
*3D U-Net++*	0.8784	5.98	0.8712	0.8857
*Attention 3D U-Net*	0.8598	6.24	0.8582	0.8613
*3D Res-U-Net*	0.8511	6.48	0.8425	0.8599
*3D DeepLabV3*	0.8442	6.59	0.8389	0.8496
*3D PSP-Net*	0.8347	6.90	0.8301	0.8394

**Table 2:** Complexity of models used in comparison process.

*Methods*	*Training Time (h)*	*Prediction Time (s)*	*Parameters*
*Our method*	26.4	55.7	52.3 mln
*3D U-Net*	15.2	76.2	19 mln
*3D Res-Net*	24.4	65.1	46.4 mln
*SwinBTS*	13.2	58.7	16.3 mln
*VT U-Net*	15.8	59.8	18.4 mln
*3D U-Net++*	25.6	64.5	51.6 mln
*Attention 3D U-Net*	18.3	121.4	22.3 mln
*3D Res-U-Net*	29.2	69.3	58.6 mln
*3D DeepLabV3*	35.8	78.6	76.1 mln
*3D PSP-Net*	36.5	96.9	72.4. mln

**Table 3:** Evaluation results for model with and without extra modules.

*Methods*	*Dice*	*Hausdorff*	*Recall*	*Precision*
*Our final result*	0.8974	5.76	0.8863	0.9087
*No pretrained weights*	0.8854	5.90	0.8784	0.8925
*No skip connections*	0.8615	6.04	0.8532	0.8699
*No attention*	0.8595	6.21	0.8548	0.8642
*No MobileNet*	Out of memory			

## Data Availability

Not applicable.
